# Electron microscopy for ultrastructural analysis and protein localization in *Saccharomyces cerevisiae*

**DOI:** 10.15698/mic2015.11.237

**Published:** 2015-10-12

**Authors:** Andri Frankl, Muriel Mari, Fulvio Reggiori

**Affiliations:** 1 Department of Cell Biology, University Medical Center Groningen, University of Groningen, Groningen, The Netherlands.

**Keywords:** electron microscopy, electron tomography, immunolabeling, chemical fixation, cryo-immobilization, correlative light and electron microscopy, Saccharomyces cerevisiae

## Abstract

The yeast *Saccharomyces cerevisiae* is a key model system for studying of a multitude of cellular processes because of its amenability to genetics, molecular biology and biochemical procedures. Ultrastructural examinations of this organism, though, are traditionally difficult because of the presence of a thick cell wall and the high density of cytoplasmic proteins. A series of recent methodological and technical developments, however, has revived interest in morphological analyses of yeast (e.g. 1,2,3). Here we present a review of established and new methods, from sample preparation to imaging, for the ultrastructural analysis of *S. cerevisiae*. We include information for the use of different fixation methods, embedding procedures, approaches for contrast enhancement, and sample visualization techniques, with references to successful examples. The goal of this review is to guide researchers that want to investigate a particular process at the ultrastructural level in yeast by aiding in the selection of the most appropriate approach to visualize a specific structure or subcellular compartment.

## INTRODUCTION

The yeast *Saccharomyces cerevisiae* is an invaluable model system for the investigation of many biological processes but also for certain ultrastructural aspects of the eukaryotic cells. It is perhaps one of the most widely employed model organisms for research in life sciences disciplines because of its amenability to genetic and biochemical approaches. By studying the yeast counterparts of mammalian proteins *S. cerevisiae* helped to determine the function of countless proteins important in human biology. As genomic projects continue to provide increasing amounts of high throughput datasets about the potential regulation and function of genes, the challenge is to assign a molecular role to the corresponding gene products and determine their overall contribution to the cell physiology. For this goal, researchers take advantage of a multitude of experimental approaches and methods. One of them is electron microscopy (EM), which allows the analysis of the ultrastructure of cells and tissues, and also of purified subcellular compartments. EM helps to study cellular processes such as cytoskeleton organization, transport vesicle formation and the establishment of organelle architecture. It also contributes to the precise localization of proteins and other cellular components. Ultrastructural EM methods rely on microscopes that use electrons to obtain images at a higher resolution than those generated by microscopes. This is due to the fact that the wavelengths of electrons are much shorter than those of the photons used by light microscopes, and consequently the resolving power is much better (up to 10 Angstrom versus approximately 200 nm).

In the past decade, innovations and breakthroughs turned EM from a mainly pure morphological approach to a much broader one, especially through integration of a variety of immunocytochemical and correlative light-electron microscopy techniques. In addition to their high resolution, another unique aspect of EM methods is that they provide information about the cellular context of the structure of interest, which very often cannot be explored with other experimental approaches. This advantage becomes even clearer when analyzing mutant cells, for the reason that EM can provide clues about the possible function of a protein and the effects caused by its mutation. The combination of EM and yeast genetics, which easily permits the knockout of a gene or the generation of point mutants, has great investigative potentials.

**Figure 1:**
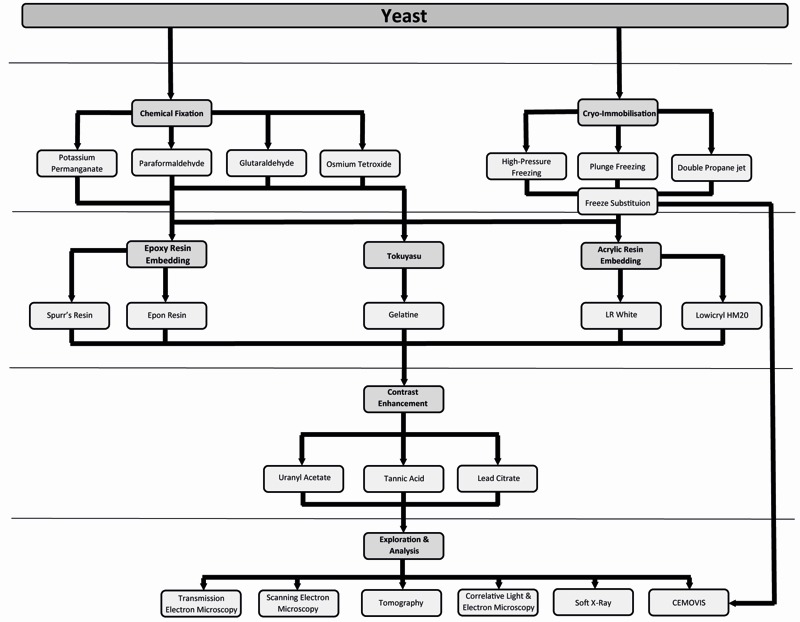
FIGURE 1: EM approaches to explore yeast ultrastructure and immunocytochemical localization of proteins. Schematic representation summarizing mainstream approaches for EM and IEM (immunoelectron microscopy) to explore the morphology and protein localization in yeast.

This potential, however, has only been minimally exploited mainly because yeast represents a challenge for most EM procedures. It possesses a cell wall, which impairs cell infiltration with chemicals and resins, and its high protein concentration in the cytoplasm, which makes it difficult to obtain good contrast and morphological resolution. Nevertheless, a series of recent EM developments and adaptations started a new era for ultrastructural investigations in this organism. Although, there is a myriad of different EM techniques (Figure 1), ranging from sample preparation to image analysis, it is often difficult to decide which could be the most appropriate approach to answer a specific biological question.

We describe and discuss techniques that have been successfully applied for yeast, and provide information to select the optimal EM method for specific research questions. While this compendium is focused on *S. cerevisiae*, most of the presented approaches are applicable to other unicellular yeast such as *Schizosaccharomyces* *pombe, Candida albicans *and *Pichia pastoris*, and they are also valid for the analysis of filamentous fungi.

## METHODS FOR YEAST CELL FIXATION

The primary goal of sample fixation is to immobilize cellular structures in a way that they remain preserved as close as possible to their native state inside a living cell. To achieve this goal a number of chemical and physical fixation methods are available. Every fixation method comes with its own advantages and disadvantages, which have to be considered depending on the goal of the analysis (Table 1). Fixation is frequently followed by the removal of water, which requires that cellular structures are stabilized sufficiently to prevent their extraction along with the water. This is done through dehydration, usually performed by using organic solvents such as ethanol, methanol or acetone. The water removal from the sample is necessary to allow subsequent infiltration with a structural support, often a resin, which can be polymerized to provide rigidity to the specimen to endure the electron beam in the electron microscope. This last step is also essential to obtain solid blocks of cells that can be easily cut and stained for EM. This general approach is commonly considered the conventional EM procedure for sample preparation.

**Table 1:** Advantages and disadvantages of different types of fixation.

**Fixatives**	**Advantages**	**Disadvantages**
Glutaraldehyde (GA)	- Irreversible fixation of proteins. - Slow penetration through the cell wall. - Some preservation of antigenicity.	- Fixation artifacts: volume changes, denatured components lead to texture changes, transformation of protein gels into reticulated structures, spatial changes due to cross-linking of proteins. - Changes in molecular bonds, i.e. creation of new bonds between macromolecules can lead to reactive site misinterpretation during labeling.
Paraformaldehyde (PFA)	- Fast penetration through the cell wall. - Preserves antigenicity better than GA.	- Causes fixation artifacts: volume change, denatured components lead to texture changes, transformation of protein gels into a reticulated structures, spatial changes due to cross-linking of proteins.
Potassium permanganate	- Fixation by oxidation of proteins and lipids. - Fast penetration through the cell wall. - Provides membrane contrast.	- Loss of fine ultrastructure. - Loss of antigenicity.
Vitrification methods (HPF, plunge-freezing, propane jet, clamp)	- Instantaneous fixation at near native state. - Well-preserved morphology and antigenicity.	- Low of contrast. - Physical damage from ice crystal nucleation. - Often requires experience and training. - It can only be applied to process a small-size samples.
Osmium tetroxide	- Rapid and irreversible fixation of proteins and lipids. - Provides pronounced membrane contrast.	- Loss of antigenicity. - Transformation of membrane phospholipids into thick unbroken lines. - Highly toxic.

The most common form of fixation for yeast is the chemical cross-linking of proteins and lipids. When performing immunoelectron microscopy (IEM) this type of immobilization is generally limited to aldehydes because they minimally alter epitopes. Fixation is generally followed by either osmium tetroxide or potassium permanganate treatments when using conventional embedding procedures with epoxy resins (see below). Chemical fixation is not an instantaneous process and consequently specific organelles such as vacuoles, which are mostly composed of water, require time to be completely immobilized and thus their morphology is frequently altered from its native state.

Due to the chemical reactions taking place during fixation, a release of protons often changes the pH, which may impede optimal cross-linking and may also affect the subsequent embedding of the sample 4. To overcome this problem, fixatives are often delivered in buffered solutions and the most frequently employed ones to maintain the neutrality are phosphate, cacodylate or PIPES buffer 5. PIPES and cacodylate buffers enhance membrane preservation via addition of calcium ions 6. While cacodylate buffers are based on arsenic, PIPES is not. It is suggested that organic buffers such as PIPES and PHEM improve cell preservation and limit the formation of electron dense precipitates compared to non-organic buffer including phosphate and cocadylate 7,8,9,10. Each buffer, in combination or not with other cations, can give a better ultrastructural preservation of specific structures 4,11.

The yeast cell wall is a significant obstacle for optimal fixation. This structure, which surrounds the plasma membrane, is a rigid extracellular polymer composed of mannoproteins, glucans and chitin 12. The reduced porosity and cross-reactions between the components of the cell wall and the added reagents make yeast cell infiltration with embedding mixtures slow and inefficient 10,13. The enzymatic removal of the cell wall with glucanases such as glusulase, lyticase or zymolyase, prior to fixation reduces these problems but could affect the cell physiology 6,14. An alternative is incubation with sodium metaperiodate, a step that can be introduced after fixation 13. Metaperiodate breaks glycosylic bonds to release proteins from the cell wall, leaving it more permeable to viscous embedding solutions.

### Aldehydes

Glutaraldehyde (GA) is one of the most common chemical fixatives used for EM and it is also employed extensively for yeast 6,10. It irreversibly binds with amino groups, like those on lysine residues, and forms various intra- and inter-protein bridges. Although GA is able to react with other molecules such as specific carbohydrates and amide groups, this compound does not bind well to lipids and therefore it is often combined with another fixative that cross-links with lipids with higher efficiency 15. One of the chemicals used for this aim is paraformaldehyde (PFA). Moreover the small size of PFA permits its rapid diffusion across the cell wall 16. GA, as a five-carbon compound, is relatively large and uncharged in a solution, and therefore its diffusion through the yeast cell wall is slower 6.

In combination, PFA initiates stabilization of cellular structures until GA can begin to react. PFA targets similar amino groups as GA but as a fixative, it is relatively unstable and consequently its cross-linking is reversible. The end result of a combination of the two fixatives is a clear image with little or no extraction, although not always perfectly accurate due to some of the possible artifacts that come from chemical fixation such as breaks, kinks or blisters in membranes. Typically, both GA and PFA are used in concentration ranging from 0.05% to 5% 4,6, with PFA in higher concentrations than GA. The irreversibility and high cross-linking properties of GA lead to severe alterations of epitopes and consequently the concentration of this chemical must be kept to a minimum if immunocytochemical examinations are planned. Other aldehydes have also been used in yeast such as acrolein 17, which is often employed together with GA, as well as other chemicals including imidoesters and peroxydisulphates 11. Currently these fixatives are rarely used especially because they do not preserve the morphology better than GA and/or PFA.

### Potassium permanganate

Potassium permanganate (KMnO_4_) was one of the first fixatives to be used for EM 18. Potassium permanganate is also a common post-fixative for yeast because unlike aldehydes, it better preserves lipid bilayers and it is thus employed alone or in combination with aldehydes 6. Permanganate binding to lipids already provides some membrane staining. The overall membrane morphology with this type of fixation appears highly contrasted, however, closer observations show an extracted morphology. Although some prominent non-membranous structures, such as ribosome and microtubules, are not preserved and certain organelles like mitochondria and lipid droplets have a partially altered morphology, other subcellular compartments such as the endoplasmic reticulum (ER), nucleus, plasma membrane, vacuole, Golgi and endosomes are well defined in permanganate-fixed yeast preparations 6,19,20,21. Potassium permanganate has been employed in a variety of concentrations in yeast, ranging from 0.5% to 6% 6 but this type of fixation is incompatible with immunolabeling due to heavily altered epitopes.

### High-pressure freezing (HPF) and freeze substitution (FS)

The use of conventional chemical fixation can sometimes lead to artifacts. Therefore physical immobilization approaches have been implemented and most of them are based on rapid freezing of the sample. There are numerous ways to cryo-immobilize yeast and the major advantages of all these methods is that they are generally instantaneous and faster than conventional chemical fixation. These methods include plunge 22, impact 23, double-propane jet, self-pressurized freezing 24 and high-pressure freezing 25. Their central principle is to vitrify (freeze the water without ice crystals formation) cells before further fixing them using chemicals. We will exclusively discuss HPF and FS because these are the most frequently used techniques, information on other types of quick freeze procedures can be found in other reviews 26,27.

HPF is currently the main approach for physical immobilization of yeast. Although other techniques such as plunge freezing and impact immobilization were more popular in the past, HPF is more reliable and efficient. Although it should be noted that it requires sophisticated and expensive equipment, this technique allows freezing relatively large quantities of yeast without cryo-protectants. HPF is achieved through application of high hydrostatic pressure and rapid lowering of the freezing point to halt the rate of ice crystal nucleation and growth 28,29. This immobilizes the liquid milieu inside and outside the yeast in a vitreous near-native state. Although volumes of yeast up to approximately 120 mm^3 ^can be high pressure frozen, a volume around 10-20 mm^3 ^is considered a more reasonable working quantity 11. Additional preservation of the native state can be achieved by adding non-penetrating cryo-protectants such as low melting point agarose or bovine serum albumin to the yeast suspension 30. These two molecules have the ability to bind water through hydrogen bonds and thereby change its freezing properties, which further aids the freezing process 31. Once the cells have undergone HPF, the water inside is extracted and substituted through a process known as freeze substitution (FS).

The principle of FS has been around for more than 40 years. The concept of dehydrating and fixing cells at very low temperatures for EM can be traced back to the 1960’s 32. FS involves substitution of the water found inside cells, initially with an organic solvent, typically acetone, ethanol or methanol, and subsequently with a resin at low temperatures before finally increasing the sample temperature to room temperature 33,34. Chemical fixatives are often added to the solvent employed for FS, to provide further immobilization during this procedure. Commonly used fixatives for FS mixtures include osmium tetroxide, uranyl acetate, PFA (0.1 - 3%) and GA (0.1 - 1%), sometime in combination with small amounts of water (0.1 - 5%) to increase membrane contrast 26.

Unlike conventional chemical fixation, the fixation steps of FS take place during or after the dehydration steps. Temperatures for FS vary between -90°C and -78°C, and the solvent will dissolve and replace the cell water over a period of hours. Fixatives are not very reactive at these low temperatures, but become homogenously distributed throughout the yeast cell despite the presence of a cell wall because of the long incubation periods (i.e. days), though rapid FS protocols have been developed as well 35. The low temperatures keep the subcellular structure in place during the diffusion and the action of fixatives. At the moment the temperature allows the fixation to occur, the fixative is already in place, homogenously distributed.

### Osmium tetroxide

This chemical is commonly used after an initial fixation with GA and/or PFA, and before embedding with a resin after FS. Osmium tetroxide binds lipids and promotes the oxidation of saturated bonds present in their fatty acid moieties causing retention of lipids in EM preparations 36. Osmium tetroxide also adds density and contrast to lipid bilayers increasing the visibility of membranous compartments. This latter characteristic is due to the molecular structure of this compound, which possesses a high density allowing electron scattering.

Unlike permanganate and aldehydes, osmium tetroxide infiltrates the cell wall of yeast even less efficiently 14. It is thus necessary to remove or permeabilise the cell wall for short incubations with osmium tetroxide to promote its penetration into the yeast cell 14. Sample incubation times with osmium tetroxide have important effects on the preparation morphology. Too short exposures do not allow a good infiltration of the yeast and a sufficient fixation of lipids, resulting in lipid extraction that is visible as blank membrane profiles on micrographs 15. Prolonged exposures, in contrast, cause the extraction of cell components especially during the subsequent dehydration steps as well as deposits of electron-dense osmium precipitates near membrane concentrations 15.

After an appropriate exposure time, between 15 and 60 minutes depending on which protocol is used, osmium tetroxide extracts cell components much less than potassium permanganate, leaving relatively small cellular components including microtubules, microfilaments, chromatin and ribosomes visible 6. Osmium tetroxide is a commonly used fixative in yeast and particularly in combination with HPF followed by FS and sample embedding in Epon or Lowicryl HM20, it has been used to investigate for example the spindle pole body and nuclear envelope 37, the cell wall structure 38, and the formation of septa and nuclear pores 39.

Osmium tetroxide fixation is not recommended when cytochemical or immunocytochemical labeling are performed because the extractions, as well as the volume and morphological changes that it causes, can lead to physical distortions that greatly interfere with the preservation of enzyme activities and antibody-antigen reactions 11. Nonetheless, in low quantities or with certain epitopes, osmium tetroxide can still be used without altering immunolabeling 22.

## EMBEDDING APPROACHES: STRUCTURAL MORPHOLOGY VERSUS IMMUNOGOLD LABELING

Yeast, just like any other cell type, have to be properly infiltrated by a chemical compound that will generate the structural support required for both sectioning and viewing in an electron microscope. In choosing the resin, one must determine primarily what the focus of the study will be: structural morphology or protein localization, i.e. epitope preservation for immunolabeling. Moreover, the chosen resin has to be appropriate to the employed fixation (see below). There are two main categories of plastic resins: epoxy and acrylic resins. Epoxy resins, such as Epon or Spurr’s, are good for resolving the cell morphology whereas acrylic resins, such as LR White and Lowicryl HM20, are better in preserving antigenicity.

### Epoxy resins

Epoxy resins initiated the age of fine structural analysis by EM; other methacrylate resins were only marginally successful 40,41. The significant advantages of epoxy resins come from their ability to cross-link virtually all structures composing a cell without losing the plasticity required to produce ultrathin sections.

**Figure 2:**
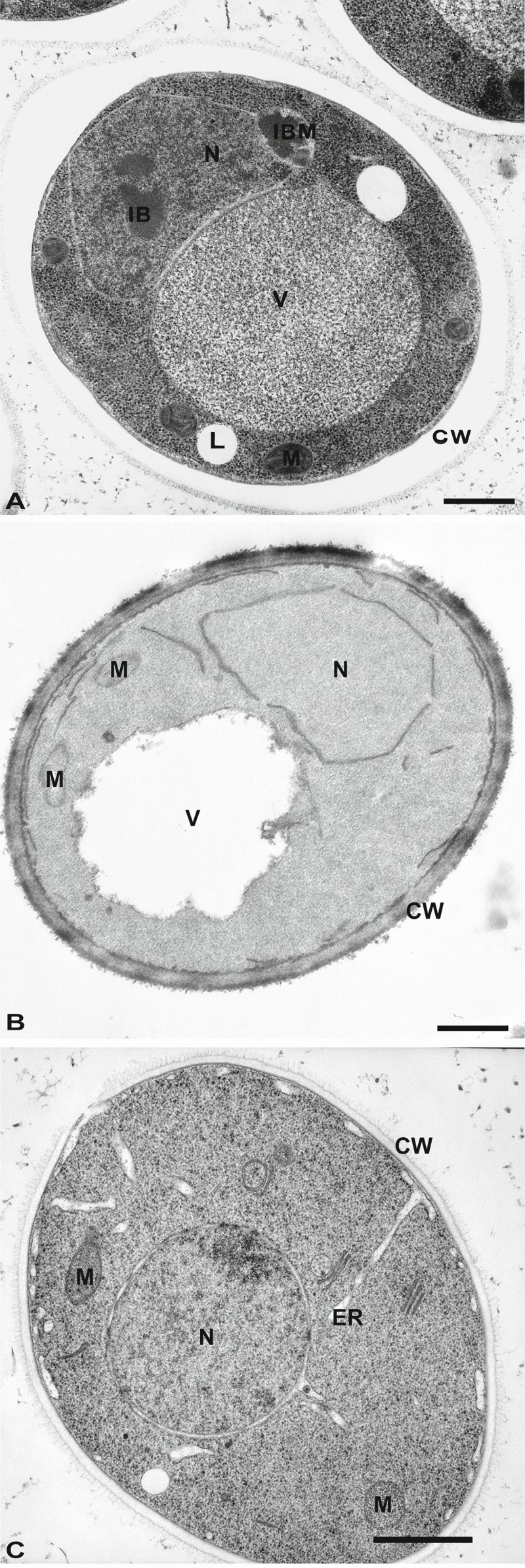
FIGURE 2: Morphology of yeast cells embedded in epoxy resins. **(A)** Cells were cryofixed in liquid propane, freeze-substituted in acetone containing 4% OsO_4_ and embedded in Epon. CW, cell wall; N, Nucleus; IB, Inclusion body; IBM, Inclusion body with membrane; L, lipid droplets; V, Vacuole. Scale bar, 0.5 µm. This image was originally published in 157 © Springer. **(B) **Yeast was fixed with 1.5% KMnO_4_, dehydrated with acetone and embedded in Spurr’s resin. CW, cell wall; M, mitochondria; N, Nucleus; V, vacuole. Scale bar, 0.5 µm. This image was originally published in 158 © the American Society for Biochemistry and Molecular Biology. **(C)** Cells were high-pressure frozen, freeze-substituted in acetone, and embedded in a mixture of Epon-Spurr’s resin. CW, cell wall; ER, endoplasmic reticulum; M, mitochondria; N, nucleus. Scale Bar, 1.0 µm. This image was originally published in 26 © Elsevier Limited.

Epon mixtures were introduced for EM in yeast in the early 1960’s 40. They continue to be used quite often as the primary embedding resin for yeast especially in combination with HPF and FS 6,25,26 (Figures 2A and 2C). It was employed to study septin rings during cell division 42 or the nucleus and vacuole connections 43. Epon resins were used in combination with potassium permanganate- or GA-fixed yeast to study for example the ER morphology 44,45. The components of a modern Epon mixture are the resin 812, the hardeners dodecenyl succinic anhydride and nadic methyl anhydride, and the accelerator benzyldimethylamine 15. Application of heat (or ultraviolet light) to this solution yields a rigid 3-dimensional polymer that is both resistant to heat and solvents, and is structurally stable under electron beams.

Another epoxy resin is the Spurr’s (or vinylcyclohexene dioxide) resin. The di-epoxide groups present in this mixture produce high cross-linking 15. Its low viscosity facilitates its infiltration into a variety of tissues that are difficult to embed including plant and yeast 46. In particular, the cell wall of yeast can be efficiently infiltrated by the Spurr’s mixture and this allows an excellent preservation of the morphology, especially that of membranous structures, such as the ER, the nuclear envelop and the plasma membrane 6. Excellent results have been obtained in combination with potassium permanganate (Figure 2B) to investigate the compartments of the secretory pathway, but also the morphology of mitochondria 47,48, vacuoles and autophagosomes 49, and the endocytic intermediates 50. Spurr’s resin has also been used to study compartments of the secretory and endosomal systems in cells fixed with either GA (followed by enzymatic removal of the cell wall) or plunge freezing 51,52,53,54,55 (Figure 2C).

As mentioned above, both Epon and Spurr’s resins allow excellent resolution of yeast morphology in combination with the appropriate fixation methods. Epoxy resins, however, present a major disadvantage because they are typically not compatible with immunocytochemical reactions aimed at localizing proteins. Due to their extremely hydrophobic nature, samples have to be completely dehydrated at room temperature or above, using solvents that can often cause protein denaturation. Furthermore the high polymerization temperatures (above 50°C) lead to further epitope denaturation. This limitation can be circumvented when studying the endo-lysosomal system through pre-embedding labeling 56 but this approach has not been applied to yeast.

### Acrylic resins

**Figure 3:**
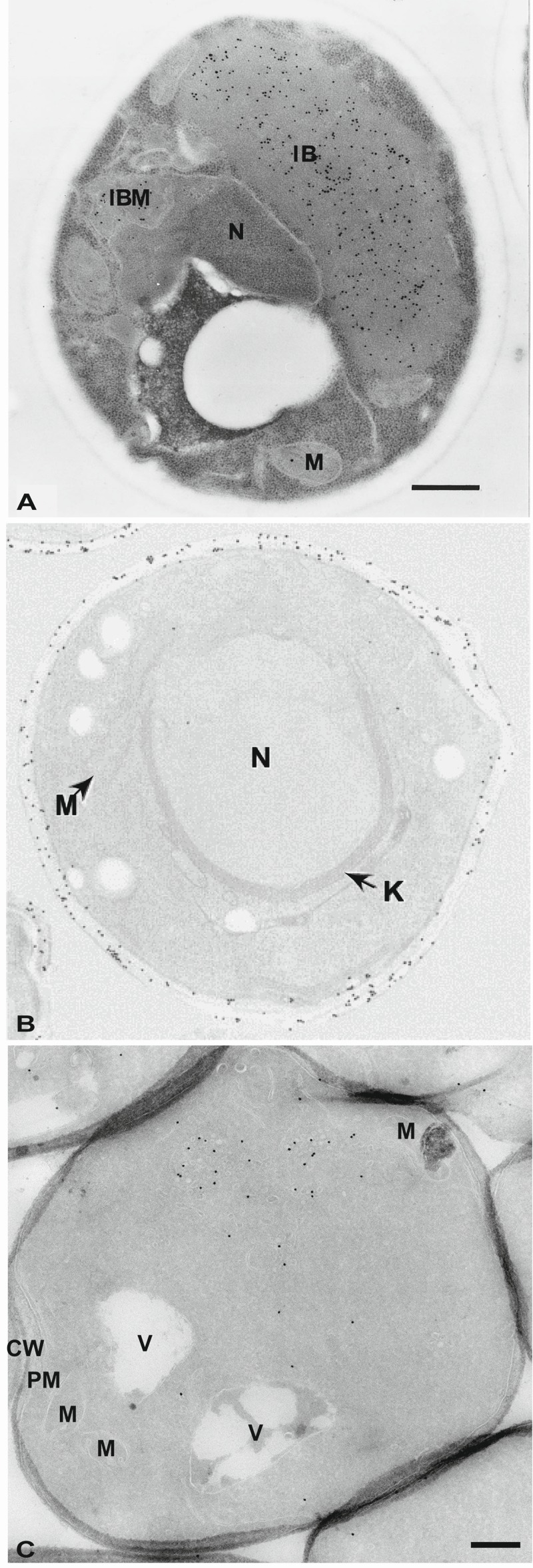
FIGURE 3: Morphology and immunolabeling of yeast cells embedded in acrylic resins or processed following the Tokuyasu method. **(A)** Yeast was cryofixed in propane, freeze-substituted in acetone containing 3% GA and embedded in Lowicryl HM20 at low temperatures. Specific antibodies and protein A were used to localize the COX complex. IB, inclusion body; IBM, inclusion body with membrane; M, mitochondria; N, nucleus. Scale bar, 0.5 µm. This image was originally published in 157 © Springer. **(B)** Cells were fixed in GA/PFA, dehydrated with ethanol and embedded in LR White resin. Immunolabeling was directed to cell wall antigens. K, karmellae; M, mitochondria; N, nucleus. This image was originally published in 6 © John Wiley and Sons. **(C)** Cells were fixed with 4% PFA and 0.4% GA, treated with sodium metaperiodate, embedded in 12% gelatin and infiltrated with 2.3 M sucrose before being frozen in liquid nitrogen. Atg9 was localized with antibodies and protein A-gold. CW, cell wall; M, mitochondria; PM, plasma membrane; V, vacuole. Scale bar, 0.5 µm. This image was originally published in 159 © Mari *et al*, 2010.

Limitations of the epoxy resins for immunocytochemical studies prompted a dedicated attempt to create more hydrophilic resins that combine good cutting properties and electron-beam resistance. A major breakthrough came with the introduction of Lowicryl mixtures like e.g. Lowicryl HM20, which are partly hydrophilic and consequently efficiently penetrate tissues 11. A mixture of aliphatic acrylates and methacrylate esters composes Lowicryl resins, which have a low viscosity and therefore they efficiently infiltrate the cell wall of yeast 11. Lowicryl HM20 was developed in 1986 57 initially to be able to handle much lower polymerization temperatures required (below -50°C) for HPF-FS approaches. Importantly, it appears that any remaining water acts as a support agent at low temperatures to stabilize protein conformation during the dehydration process 58. The fact that lower temperatures significantly decrease the negative effects of dehydration on structural preservation and epitope denaturation, as well as negligible lipid extraction, makes immunolabeling reactions on Lowicryl resin-embedded samples more effective 59,60. In yeast, Lowicryl HM20 has been employed in combination with either chemical (Figure 3A) or HPF fixation to immunolocalize for example vacuolar enzymes 13 or proteins accumulated in the ER 61.

Another polyhydroxy-aromatic acrylic resin is LR White 62. This low viscosity mixture requires tissue dehydration before infiltration and allows a rapid embedding. Its polymerization can be initiated in different ways, i.e. by either heating to temperatures above 50°C, exposure to UV or addition of an aromatic tertiary amine that accelerates chemical reactions. LR White resin has also been used for a number of studies using chemically (Figure 3B) or physically fixed yeast to localize through immunological reactions nuclear pore complex subunits 63, plasma membrane Gas1 64, endosomal proteins 65,66, endocytosed factors 67, actin 68, ER 69 and spindle body components 70.

### The Tokuyasu method

The thawed-frozen section technique is better known as the Tokuyasu method, from its developer’s name 71,72. This approach utilizes ultra-thin sections that are obtained by cryo-ultramicrotomy from material that has been chemically fixed by aldehydes, embedded in gelatin and frozen in liquid nitrogen. Immunolabeling and imaging of the sections, however, are done at room temperature. This technique has many advantages over other embedding procedure because it provides a high-resolution of membranes as well as a higher efficiency of immunological reactions 73. The Tokuyasu method remains one of the most sensitive post-sectioning techniques for immunolabeling because aldehyde fixation is the only denaturing step for antigens (i.e. samples are not treated with organic solvents).

This method has recently been optimized for yeast 74 (Figure 3C). The major modification in the protocol has been the introduction of a post-fixation treatment with metaperiodate to promote an infiltration of gelatin. Since cryo-sections obtained with the Tokuyasu method are not contrasted using a negative staining but rather with uranyl acetate and lead citrate (see below), the extraction of the non-optimally fixed lipids and the high protein concentration in the cytoplasm create negative contrast that leads to a unique resolution of the yeast morphology 74.

The Tokuyasu method adapted to yeast has been successfully used to perform localization studies on mitochondria 75,76, endosomes 77,78, subdomains of the plasma membrane 79, nuclear pores 80 and autophagosomal membranes 81,82,83. Lipids tend to be extracted during the preparation of cryo-sections because they are not optimally fixed and therefore structures like lipid droplets with membranes low in protein concentrations are not optimally preserved. A way to overcome this problem and other possible fixation artifacts is that the Tokuyasu technique is not restricted to chemical fixation but it can also be combined with physical immobilization by HPF followed by FS and a rehydration step 84. This approach appears to work with yeast samples as well 74.

## MEMBRANE CONTRASTING METHODS

The gun of an electron microscope emits a beam of electrons with a particular wavelength that depends on the acceleration voltage applied. A phosphor-coated screen makes the electrons passing through sections visible by absorbing them. This results in an image being drawn by the density of the sample staining and the resulting intensity (number) of electrons hitting the phosphor-coated screen. The components and structures present in biological samples have generally very little differences in density and consequently the contrasts in the image formed are minimal. Therefore, it is important to increase contrast in the sample (Table 2). This can be achieved by increasing the densities of structures by binding heavy metal salts to them. There are two main approaches to stain EM samples with heavy metals: (i) positive staining exhibits a positive contrast by increasing the density of a particular biological structure rather than any contiguous surrounding area; (ii) negative staining through heavy metal salts increases the density of the area around a specific molecular structure so that the structure of interest appears lighter than the surrounding material. It must be noted that there are also contrasting methods such as tannic acid (TA) staining that do not rely on heavy metal salts 85. Generally staining is carried out once sections have been cut and any immunocytochemical labelling has been performed.

**Table 2:** Combinations of embedding media, fixation methods and staining procedures employed for yeast ultrastructural analyses.

	**Epon**	**Spurr's**	**LR White**	**Lowicryl HM20**	**Tokuyasu preparations**
***Fixation***					
Glutaraldehyde	(X)	(X)	X	X	X
Paraformaldehyde	ND	(X)	X	X	X
Potassium permanganate	ND	X	ND	ND	ND
HPF/FS	X	(X)	(X)	X	(X)
***Staining***					
Osmium tetroxide	X	(X)	ND	X	ND
Uranyl acetate	X	X	X	X	X
Lead citrate	X	X	X	ND	(X)
Tannic acid	X	ND	ND	(X)	ND

### Positive staining

The two most common compounds used for the positive contrasting are uranyl acetate (UA) and lead citrate (LC). The staining mechanisms of these chemicals are not completely understood. Uranyl ions may be strongly attracted to phosphate and specific amino groups, which facilitates the identification of nucleic acids and proteins 86. In contrast it is thought that lead ions bind to mostly negatively charged molecules such as hydroxyl groups or areas that have reacted to osmium tetroxide 87. UA and LC are thus considered non-specific as they stain numerous different cellular components 15 and because of their complementary reactivity, they are often employed in combination to obtain better contrast. UA and LC are compatible with all the types of fixation and sample embedding, and the vast majority of EM analyses of yeast but also other organisms use these two heavy metal salts to contrast membranes.

### Negative staining

Negative stains are often made from heavy metal salts such as uranyl, tungsten or molybdenum 15. The heavy metal staining does not affect the macromolecular structures themselves, as with positive staining procedures, but rather the surrounding area. This results in a specimen that appears to be in negative contrast, i.e. a lighter tone against a darker background 15. Although commonly used to identify small structures such as viruses, bacteria or little organelles, it can also be employed for the analysis of organisms of small size such as yeast 88. The major advantage of negative staining compared to positive staining is that it highlights the structure of interest, especially when of small dimensions, without staining the structure itself, something that could alter its fine ultrastructural details. This aspect has been exploited in yeast to study glucan polymer formation during the regeneration of the cell wall in protoplasts 21,89 and protein filaments 90.

### Tannic acid staining

Since it was first utilized as a mordant, i.e. a chemical that both fixes a dye on a cellular component and forms an insoluble compound with the dye, TA has become widely spread in its use because it optimally fixes a variety of different tissues and cells either by itself or in conjunction with GA 91. TA in particular binds with high affinity to collagen, glycogen and various other subcellular complexes. Although it acts as a fixative, its mordant properties are very useful to greatly enhance the sample contrast 85. As one of the few alternatives to the use of heavy metal salts, TA appears to avoid regions that would be stained by conventional contrasting agents such as UA. Therefore its use, alongside other staining agents, provides different contrasting patterns depending on the combination. In yeast, TA has only marginally been employed mostly as a post-embedding contrasting agent, to analyze purified microtubules 92 and nuclei 93 and COPII-coated vesicles 94.

## 2D AND 3D VISUALIZATION TECHNIQUES

Once the yeast samples are prepared, a variety of different imaging methods that revolve around the basic principles of EM are available, from widespread and fundamental approaches like transmission electron microscopy (TEM) and scanning electron microscopy (SEM), to more sophisticated techniques like correlative light-electron microscopy (CLEM), electron tomography (ET), cryo-electron microscopy of vitreous sections (CEMOVIS) and soft X-ray tomography. The analysis method largely determines the procedure of sample preparation and the type of data that are extracted from the sample. Each technique has its own strength and weakness and it is wise to carefully consider the research goal before opting for a particular approach.

### Transmission electron microscopy

TEM is the most commonly employed form of EM, it has a resolution hundreds of times higher than that of the classical light microscopes and consequently it can visualize macromolecular structures and organelles that compose the cell at the nanoscale level. It consists of an interconnected set of electromagnetic lenses that channel a beam of primary electrons towards the sample 15. As the primary electrons pass through the sample, they create a two-dimensional (2D) projection image with fine structural details 6. TEM is easily handled by relatively inexperienced operators and can give some of the most detailed and high quality images that can be obtained 95. With its high magnification and resolution, TEM makes it possible to see many of the structures present inside a yeast cell 95, which are not detectable and/or identifiable through light microscopy approaches including super-resolution ones. As TEM is one of the most widely used forms of EM for biological samples, a vast variety of publications are available. A large number of them are about the morphological and functional characterization of subcellular compartments of yeast processed for EM using preparation obtained with practically all the procedures presented in this review.

### Electron tomography

ET is a method that generates three-dimensional (3D) reconstructions of a cellular structure, which provide more thorough and complete insights into its organization and possible functions. As a conventional electron micrograph has a large depth of focus and generates 2D projections, features in the z-axis of the section are superimposed on top of each other, making it hard to analyze and interpret them especially in thicker sections 96. The improved insights are generated from a z-axis resolution that is at least 10 times better than the one of the average 2D projection image.

Initially an approach called serial sectioning was developed to introduce the third dimension in TEM, which has also been employed for studies in *S. cerevisiae *fixed with permanganate, GA/PFA or HPF before being infiltrated with an epoxy resin 97,98,99,100,101,102. This technique involves the collection of several successive serial sections of the same sample, and then superposes and aligns their 2D images to generate 3D models. Some disadvantages for serial sectioning include a loss of material when handling cryo-sections and the stitching of multiple 2D projections can be very difficult. ET overcomes most of these issues and has the additional advantage of a simpler image collection and model reconstruction routine as well as a higher z-axis resolution. This technique combines a higher electron output with a tilt series of images created by rotating the specimen holder incrementally around a fixed axis 103. The obtained tilt series of images (i.e. a collection of a large number of 2D images) are then stacked together and converted into a 3D representation of the sample.

ET has many of the same strengths and weaknesses as conventional TEM, but it is able to create a 3D image with a 1 - 10 nm resolution, which is similar to that of TEM (0.1 - 1 nm) and SEM (1 - 10 nm) 95. It must be noted, however, that the 3D reconstructions created from ET tilt series of images are not complete representations. This is due to the limitation of the microscope sample holder that makes only possible to tilt the sample to a maximum of 60-70 degrees. This leaves the reconstructions with undefined cone shaped areas and consequently ET does not provide a complete 360 degrees overview of the zone of interest.

Alongside conventional TEM, ET has become a very prominent approach in many areas of cell biology and has been inclemently introduced in investigations performed in yeast as well. The most frequent approach has been to fix yeast through HPF and embedding in Epon, Spurr’s or Lowicryl HM20 resins before performing ET on thick sections that can range from 0.2 to 1 µm 21,26,104. This type of methodology has, in between others, allowed studying the mitotic spindle/nuclear envelop 105,106, the septin rings formed between two dividing cells 42,107, multivesicular body formation 108,109,110, various aspects of mitochondrial ultrastructure 111,112,113, plasma membrane reshaping during endocytosis 114, and ER morphology 111,113. Chemical fixation with permanganate followed by embedding in Epon resin has also been successfully used for electron tomography studies of lipid droplets 115. Recently, 200 - 250 nm serial cryo-sections obtained with the Tokuyasu methods were resolved by ET and through immunolabeling proteins were localized in 3D reconstructions 1,75.

### Scanning electron microscopy

SEM was developed approximately at the same time as the TEM. SEM can directly collect 3D representations of a cell surface or even an entire specimen but with lower resolution than TEM. SEM uses a focused de-magnified spot of electrons to scan over an electrically conductive specimen. The result of the electrons hitting the specimen is the release of a number of signals such as secondary electrons, backscatter electrons and X-rays 15. Sensitive detectors that are specifically created for detecting them collect these various signals.

SEM is limited in terms of resolution at a high magnification when compared to conventional TEM (1-10 nm versus 0.1-1 nm, 95) and thus it is usually employed to acquire information about the topology and morphology of the sample surface, rather than the internal morphology of a cell obtained by TEM. As a result, SEM is generally not used for EM-based immunolocalization studies but this is slowly changing with the introduction of new protocols and equipment 116. However, there are a number of techniques that can be coupled to SEM to provide additional information. An example of these methodologies that is applied for the analysis of yeast is the focused ion beam-scanning electron microscopy (FIB-SEM), which allows the construction of 3D representations (with lower resolution than those ET), but permits the employment of much larger samples, up to 1 µm 117,118. FIB-SEM has been exploited to generate 3D reconstructions of whole yeast cells, fixed with either GA/PFA and permanganate or HPF, before being embedded with a resin 119,120,121,122.

It should be noted that most biological specimens must be thoroughly dehydrated (i.e. critical point drying) and covered with a conductive metallic support film before being imaged by SEM. These treatments can distort cellular features and cause artifacts 15, though this does not apply to most embedded and sectioned samples due to the lower mass of specimen. There are several studies that relied on SEM of fixed and subsequently dehydrated yeast cells to examine surface features, i.e. cell wall and plasma membrane 123,124,125,126, but also cellular components like the nuclear pore complex is studied on isolated nuclei 93.

### Correlative light-electron microscopy

The term CLEM includes all those methods that exploit light microscopy to localize structures of interest and subsequently determine ultrastructural details by EM (reviewed in 127,128,129,130). These methods provide further insight into specific protein localizations that cannot be obtained by standard IEM because the immunological reaction does not allow the detection of the protein of interest 127. Another application is the ultrastructural identification of a particular fluorescently labeled structure being monitored by fluorescent imaging. These latter approaches often require the fusion of the studied protein with a tag such as the green fluorescence protein (GFP), which can be visualized by fluorescent microscopy 131. Subsequently, GFP is directly detected on the EM preparations if the employed fixation method and embedding support do not alter its ability to emit fluorescence upon excitation.

While some proteins have been optimized to retain their fluorescence capacity after EM-preparation 132, successful approaches employed fixation by either HPF followed by CEMOVIS (see below, 133,134,135) or FS embedding in Lowicryl HM20 2,114, or plunge-freezing before application of the Tokuyasu method 136. Alternatively, GFP can be indirectly localized on EM preparations through either immunolabeling or chemical reactions if the tag consists of GFP fused with an enzyme that generates an electron dense precipitate, such as in the FLIPPER tag 3. CLEM techniques have become very popular during the last decade and some have been applied to yeast studies. An example is the localization and characterization at the ultrastructural level of Sup35 prions with very good morphological results 137. For this analysis yeast has been fixed with GA before being first imaged by fluorescence microscopy, and then processed for EM after OsO_4_ post-fixation and embedding into Epon812. Another example has been the analysis of actin filaments using the marker protein GFP-Abp1 131.

Importantly, HPF and subsequent yeast cell embedding with Lowicryl HM20 allows to preserve the fluorescence of GFP in section and together with a new tool to correlate the fluorescence signal to EM preparations, this approach has successfully been used to study early endocytic events 2. Tomographic CLEM analyses can also be performed on thick cryo-sections obtained using the Tokuyasu method and labeled then with antibodies conjugated to a fluorescent group 75. Finally, CEMOVIS and soft X-ray tomography have also been employed for CLEM in yeast (see below).

### Cryo-electron microscopy of vitreous sections

CEMOVIS (or cryo-EM/tomography) is a technique that employs vitrified biological samples and allows the observation of the specimen in a near native state 138,139,140. Samples can range from cryo-sections of different thickness to entire cells if their width does not exceed 0.5 - 1 µm, and can be analyzed by TEM, SEM or ET. Moreover the serial sectioning of vitreous samples permits obtaining 3D reconstructions of larger samples 141. The fact that samples are at a near native state infers that those chemicals, which could alter some ultrastructural details, have not been used 95.

CEMOVIS has, however, some downsides. Vitrified samples are unstained and therefore the contrast gained during the preparations of the sections is extremely low 95. Because of this limitation and the fact that CEMOVIS requires sophisticated equipment (HPF system, cryo-ultramicrotome, cryo-EM, cryo-holder for ET…) as well as a high degree of technical expertise, published works exploiting this approach are scarce but are steadily increasing especially due to the major availability in HPF technologies, which overcome the previous use of plunge or slam freezing for sample vitrification. So far yeast has been exclusively used for proof-of-principle demonstrations for methods to be applied with CEMOVIS 133,134,135, but the resolution degree shown is very promising. Another disadvantage of CEMOVIS is that frozen preparations cannot be immunolabeled, which limits localization studies. Fluorescently tagged fusion proteins, however, are optimally preserved and as a result fluorescence signals can potentially be used for CLEM investigations 142.

### Soft X-ray tomography

This method combines the features of light and electron microscopy. It is an easy and high throughput technique (similarly to light microscopy) that allows collecting low-resolution, absorption-based images similarly to EM 143. Soft X-ray tomography permits a user to view a whole hydrated cell, and to examine its morphology at a high spatial resolution (0.8 µm) up to 15 µm deep. It is based on the principle of X-rays being absorbed directly by the different cellular components and the resulting image is practically a projection of the dose of X-rays passing through the specimen 144,145,146. Organelles inside the cell are visualized directly due to their different biochemical composition and density. For example, a compartment with a high lipid content is much more sensitive to X-rays than an organelle that contain a significant portion of water such as the vacuole 147.

One of the big advantages of the soft X-ray tomography is its circumvention of the use of potentially damaging fixation and staining procedures because samples are cryo-immobilized. Another positive consequence of this feature is that this technique also allows performing CLEM examinations through the determination of the subcellular distribution of molecules tagged with a fluorescent label in entire and intact yeast cells projected in 3D 148. On the downside of this approach is the relatively low resolution of the images, 50 nm at the maximum, which can vary depending on the machine used and the analyzed structure 149. The 3D reconstructions, however, are accurate. While soft X-ray tomography requires special software and a very sensitive machinery able to detect the X-rays passing through the yeast samples, this technique is very helpful to determine organelle position, quantity, and structural changes due to growth conditions or mutations in large cell populations 143,147,150,151. For example this approach has been used to demonstrate that the volumetric ratios between organelles such as the nucleus, nucleoli, mitochondria, vacuoles and lipid particles do not change throughout the cell cycle 150 or identifying factors required to regulate the shape of the mitotic nucleus 152.

## CONCLUSIONS

EM has considerably contributed to the field of biological sciences over the past 60 years. Virtually every organelle and major structure of the cell has been discovered and characterized by EM. This has allowed researchers unraveling morphological details of healthy cells and the changes that they undergo in diseased or mutated states.

To determine which EM approach to use, it is crucial to consider the end goal of the research question. Choosing a particular imaging method, i.e. TEM, SEM or tomography will already reduce the available options for a researcher. As shown in Figure 1 and Table 2, there are many possibilities and no single defined route for sample preparation and analysis is universally applicable to solve all the questions. Different sample preparation steps can be combined or interchanged. For example one can employ HPF for cryoimmobilisation before either rehydrating the cells and using the Tokuyasu method or embedding them in a resin such as Epon. Therefore it is important to determine whether topographical details or conventional TEM images are required, or whether immunocytochemical methods will be employed to localize proteins. Another relevant aspect to consider is which subcellular organelle or structure will be examined.

Differences in lipid and protein composition, concentration and density can lead the cell components to be differently preserved and resolved depending on the employed fixation method, embedding support and contrasting agent. Sometimes it is better to choose a sample preparation method that is not ideal for immunological reactions but that provides a better morphology of the labeled structures rather than having a sample ideal for protein localization where the structure of interest is not clearly defined.

One of the advantages of EM approaches is that there are several alternatives to choose from. A major obstacle in combining different EM methods, however, will be the eventual availability and accessibility to a specific EM instrument.

We also wish to emphasize that ultrastructural observations must contain statistical evaluation. Rigorous stereological methods, including unbiased sampling techniques, can provide very precise quantifications about the subcellular distribution of a protein or compartment, the surface or volume of an organelle, or the recurrence of a phenotype 153,154,155. To this aim, few guidelines have to be kept in consideration when designing and realizing EM analyses. Experiments have to be performed in triplicate and countings have to be done randomly. Typically 50-100 cells per experiment have to be examined but this number has to be increased if what is counted is infrequent. When carrying out IEM, two critical controls evaluating the specificity of the used antibodies have to be included to get an accurate estimation of the relative distribution of a protein. The first is to immunolabel sections prepared from cells not expressing the analyzed protein. The second is to perform an immunolabeling reaction that does not include the primary antibody.

As important as the invention of the electron microscope was, its continuous development and the integration of other specialized techniques and hardware are what makes cellular morphology a real corner stone of modern research. As a result of ongoing developments and improvements of computer software, electron detection systems, image enhancement solutions, automated quantification, new CLEM probes, multifunctional EM machines and data storage, EM has a bright future and it will move from a relative small-scale to large throughput type of analysis. Moreover machines and procedures that have recently been developed and used in other cell types such as serial section SEM 156 and serial block face SEM 157 or CLEM tags like miniSOG 156 or APEX 158, could also be applied to yeast. These advances will also result in more objective- and quantitative- studies than ever. Ultrastructural research in yeast will also benefit from these progresses and the continuous adaption and improvement of the established and new EM protocols for other systems will make ultrastructural studies in this model system a routine approach for investigators. In addition to having a large collection of mutant and knockout strains, yeast strains provide the straightforward possibility of endogenous gene fusion with sequences encoding for protein tags 156. As a result, the development of new primary antibodies for IEM analyses is not an absolute necessity because it is sufficient to purchase a commercially available secondary antibody recognizing the tag and known to work for ultrastructural studies.
